# Headache disorders in a tertiary care headache outpatient clinic in Austria

**DOI:** 10.1186/1129-2377-14-S1-P30

**Published:** 2013-02-21

**Authors:** M Fischer, G Wille, S Klien, G Broessner

**Affiliations:** 1 Innsbruck Medical University, Austria

## Background

Headache is a common cause of medical consultation, both in primary care and in specialist neurology outpatient clinics. This study analyzed the incidence and characteristics of headache patients during an 18-month period in a tertiary care headache outpatient clinic.

## Material and methods

Patients visiting a tertiary care headache outpatient clinic at the University Hospital Innsbruck, Austria, were registered consecutively. Diagnosis of primary and secondary headache disorders was in accordance with the revised version of the International Headache Classification (ICHD-II) and data including age, sex, Headache Impact Test (HIT-6), headache frequency, trigger factors and caffeine intake were collected prospectively over an 18-month period.

## Results

From December 2010 until April 2012 626 patients comprising 897 visits were registered in our headache database. The female to male ratio was 3:1. The median age accounted for 40.7 years (IQR: 28.3 ¨C 50.4). Diagnoses of primary headache disorders were grouped as follows: migraine 61% (episodic migraine with aura 22 %, episodic migraine without aura 37%, chronic migraine 2%), episodic (12%) and chronic tension-type headache (8%), trigeminal autonomic cephalalgias (4%), other primary headaches (1%), secondary headaches (13%),cranial neuralgias and facial pain (2%) and currently unclassified (15%). The majority of patients with secondary headache disorders were diagnosed with headache attributed to a substance or its withdrawal (10%) The median number of headache days per month was 8 (IQR 4-20). 33% of all patients reported >15 headache days during the previous month.

## Conclusions

This registry reflects the characteristics of patients seen in an outpatient headache clinic. In accordance with other data from tertiary hospitals migraine was the most common diagnosis in our outpatient clinic followed by tension-type headache. Almost 33% suffered from frequent (¡Ý15 days per month) headache reflecting a substantial and growing patient population with medico-economical burden to the headache community.

